# Intravenous multipotent adult progenitor cell therapy after traumatic brain injury: modulation of the resident microglia population

**DOI:** 10.1186/1742-2094-9-228

**Published:** 2012-09-28

**Authors:** Peter A Walker, Supinder S Bedi, Shinil K Shah, Fernando Jimenez, Hasen Xue, Jason A Hamilton, Philippa Smith, Chelsea P Thomas, Robert W Mays, Shibani Pati, Charles S Cox

**Affiliations:** 1 Department of Surgery, University of Texas Medical School at Houston, 6431 Fannin Street, MSB 5.236, Houston, TX, 77030, USA; 2 Pediatric Surgery, University of Texas Medical School at Houston, 6431 Fannin Street, MSB 5.236, Houston, TX, 77030, USA; 3 Michael E DeBakey Institute for Comparative Cardiovascular Science and Biomedical Devices, Texas A & M University, College Station, TX, USA; 4 Department of Regenerative Medicine, Athersys Inc, 3201 Carnegie Avenue, Cleveland, OH, 44115, USA

**Keywords:** Multipotent adult progenitor cells, Traumatic brain injury, Stem cells, Splenocytes, Blood brain barrier, Microglia

## Abstract

**Introduction:**

We have demonstrated previously that the intravenous delivery of multipotent adult progenitor cells (MAPC) after traumatic brain injury affords neuroprotection via interaction with splenocytes, leading to an increase in systemic anti-inflammatory cytokines. We hypothesize that the observed modulation of the systemic inflammatory milieu is related to T regulatory cells and a subsequent increase in the locoregional neuroprotective M2 macrophage population.

**Methods:**

C57B6 mice were injected with intravenous MAPC 2 and 24 hours after controlled cortical impact injury. Animals were euthanized 24, 48, 72, and 120 hours after injury. *In vivo,* the proportion of CD4^+^/CD25^+^/FOXP3^+^ T-regulatory cells were measured in the splenocyte population and plasma. In addition, the brain CD86^+^ M1 and CD206^+^ M2 macrophage populations were quantified. A series of *in vitro* co-cultures were completed to investigate the need for direct MAPC:splenocyte contact as well as the effect of MAPC therapy on M1 and M2 macrophage subtype apoptosis and proliferation.

**Results:**

Significant increases in the splenocyte and plasma T regulatory cell populations were observed with MAPC therapy at 24 and 48 hours, respectively. In addition, MAPC therapy was associated with an increase in the brain M2/M1 macrophage ratio at 24, 48 and 120 hours after cortical injury. *In vitro* cultures of activated microglia with supernatant derived from MAPC:splenocyte co-cultures also demonstrated an increase in the M2/M1 ratio. The observed changes were secondary to an increase in M1 macrophage apoptosis.

**Conclusions:**

The data show that the intravenous delivery of MAPC after cortical injury results in increases in T regulatory cells in splenocytes and plasma with a concordant increase in the locoregional M2/M1 macrophage ratio. Direct contact between the MAPC and splenocytes is required to modulate activated microglia, adding further evidence to the central role of the spleen in MAPC-mediated neuroprotection.

## Introduction

Traumatic brain injury (TBI) affects nearly 1.5 million patients in the United States annually [1]. TBI is associated with significant long-term physical, cognitive, and psychosocial deficits leading to an annual economic impact of 60 billion dollars. The prevention of primary brain trauma has proven to be difficult. Therefore, a large amount of research is currently underway to investigate potential treatments that could limit the secondary injury associated with TBI. One possible pathway towards neuroprotection is via modulation of the proinflammatory environment observed after TBI.

The central nervous system (CNS) is a complex arrangement of interacting cells including glia and neurons. Microglia (five to ten percent of the total glial population) represent the resident immune cells (akin to tissue macrophages) in the CNS [2]. Early activation of microglial cells has been observed in both TBI and spinal cord injury {Chirumamilla, 2002 #27, [3], Beck, 2010 #28, [4]}.

The two citations are as below added to reference section and should be numbers 3 and 4 which means the subsequent numbers in the text for references are changed.

The number and distribution of cerebral macrophages (endogenous and exogenous) and global activation after injury make them a likely candidate effector cell population.

Following acute injury, microglia are activated and differentiate into neurotoxic proinflammatory M1 macrophages [5]. Classically activated M1 macrophages are responsible for the continued production of proinflammatory cytokines and potentially cytodestructive substances. Immediately following TBI, the ratio of M1 (proinflammatory) to M2 (an alternatively activated, anti-inflammatory subtype) macrophages is typically 1:1, but as time progresses (Day 3 to Day 7), the ratio favors the M1 subtype [6]. In addition, an initial decrease in M2 macrophages with concordant increase in M1 macrophages is observed early after spinal cord injury [7]. Alteration of the biochemical milieu after injury is known to activate microglia towards alternate activation pathways [8]. Anti-inflammatory cytokines such as interleukin-4 (IL-4) and interleukin-10 (IL-10) have been shown to lead to preferential differentiation of microglia into neuroprotective M2 subtype macrophages [8,9].

Multipotent adult progenitor cells (MAPCs) are derived from bone marrow [10] and have been associated with neuroprotection in a rodent TBI model [11]. Walker *et al*. [11] showed that the intravenous delivery of MAPCs after TBI preserved the blood brain barrier, preserved splenic mass, and increased anti-inflammatory cytokine production (IL-4 and IL-10). Further conclusions indicated that MAPCs interacted with splenocytes leading to an increase in circulating CD4+ T cells [11].

A subtype of CD4+ T cells (FoxP3+) is known to have anti-inflammatory properties. We hypothesize that the observed increase in anti-inflammatory cytokine production with MAPC therapy after TBI is secondary to an increase in the FoxP3+ T regulatory cell population. We hypothesize that the increase in anti-inflammatory cytokines previously demonstrated affects the resident microglial population leading to an increase in the M2/M1 macrophage ratio thereby accounting for the observed neuroprotection. To test our hypothesis a series of *in vivo* and *in vitro* experiments were completed, using MAPCs in a mouse model of TBI.

## Methods

All protocols involving the use of animals were in compliance with the National Institutes of Health *Guide for the Careand Use of LaboratoryAnimals* and were approved by the University of Texas Medical School at Houston’s Institutional Animal Care and Use Committee (protocol HSC-AWC-10-039).

### *In vivo* experiments

#### Experimental design

**Figure 1:**
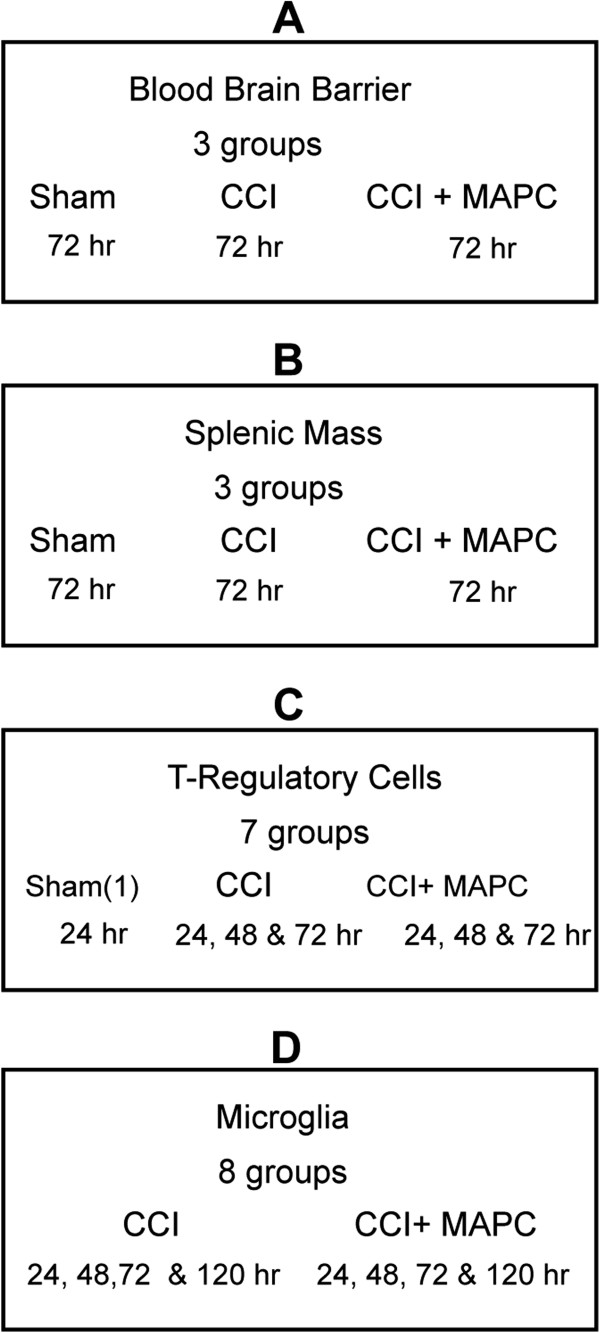
**Experimental design for *in vivo *experiments.** Four sets of experiments were done in order to determine (**A**) blood brain barrier, (**B**) splenic mass, (**C**) characterization of T-regulatory cells in spleen and blood, and (**D**) characterization of local microglia phenotype using flow cytometry.

Three groups of C57B6 mice underwent controlled cortical impact (CCI) injury (n = 6/group, 2 groups) or sham injury (n = 5). Human multipotent adult progenitor cells (hMAPC), produced under cGMP conditions that have been previously described [12,13], were provided by Athersys, Inc. (Cleveland, OH, USA). One group of injured animals had 1 × 10^7^ MAPC/kg injected via the tail vein at 2 and 24 hours after CCI injury. Seventy hours after CCI injury, Evan’s blue dye was injected into the animal via the tail vein. After 1 hour of circulation, the animals were euthanized with subsequent homogenization of the injured cortical hemisphere and overnight incubation in formamide. Blood brain barrier (BBB) permeability was determined via measurement of Evan’s blue absorbance (Figure 1A). In order to measure splenic mass, two additional groups of C57B6 mice underwent controlled cortical impact (CCI) injury (CCI alone and CCI plus MAPC) and sham injury (n = 18/group, Figure 1B).

An additional seven groups (n = 4/group) of C57B6 mice underwent controlled cortical impact (CCI) injury (six groups) or sham injury (one group). Three groups of mice received 1 × 10^7^ MAPC / kg injected via the tail vein at 2 and 24 hours after CCI injury. One CCI injury alone control group and one MAPC treated group were euthanized at 24, 48, and 72 hours, with the sham injured group being euthanized at 24 hours. At the time of sacrifice, spleens were harvested and the percentage of CD4^+^/CD25^+^/FOXP3^+^ T regulatory cells was measured by flow cytometry (Figure 1C).

An additional seven groups (n =6/group) of C57 Black 6 mice underwent controlled cortical impact (CCI) injury (six groups) or sham injury (one group). Three groups of mice received 1 × 10^7^ MAPC / kg injected via the tail vein at 2 and 24 hours after CCI injury. One CCI injury alone control group and one MAPC treated group were euthanized at 24, 48, and 72 hours, with the sham injured group being euthanized at 24 hours. At the time of sacrifice, the brain was homogenized, the cell populations were separated by density separation, and the CD86^+^ M1 and CD206^+^ M2 macrophage populations were analyzed by flow cytometry (Figure 1D).

#### Controlled cortical impact injury

A controlled cortical impact (CCI) device (eCCI Model 6.3; VCU, Richmond, VA, USA) was used to administer a unilateral brain injury as described previously [14]. Male mice weighing 17 to 21 grams were anesthetized with 4% isoflurane / O_2_ and the head was mounted in a stereotactic frame. Animals received a single impact of 1.0-mm depth of deformation with an impact velocity of 5.0 m/sec and a dwell time of 150 msec (moderate-severe injury), making the impact to the parietal association cortex. Sham injuries were performed by anesthetizing the animals, making the midline incision, and separating the skin, connective tissue, and aponeurosis from the cranium. The incision was then closed [15,16].

Preparation and intravenous injection of MAPCHuman MAPCs were obtained from Athersys, Inc. and stored in liquid nitrogen. Prior to injection, the MAPC were thawed, washed and resuspended in phosphate buffered saline (PBS). Cells were counted and checked for viability via Trypan blue exclusion (>95% viability). Immediately prior to intravenous injection, MAPC were mixed gently eight to ten times to ensure a homogeneous suspension. MAPC were injected via the tail vein at 2 and 24 hours after CCI injury at a dose of 1 × 10^7^ MAPC / kg. Therefore, each treatment animal received two separate doses of MAPC. CCI alone animals received PBS vehicle injection alone at the designated injection time points.

#### Evan’s blue BBB permeability analysis

Seventy-two hours after CCI injury, the mice were anesthetized as described above, and 0.2 mL of 3% Evan’s blue dye in PBS was injected via the tail vein. The animals were allowed to recover for 60 minutes to allow for perfusion of the dye. After this time, the animals were euthanized via right atrial puncture and perfused with 4% paraformaldehyde. Next, the animals were decapitated followed by brain extraction. The cerebellum was dissected away from the rest of the cortical tissue. The brain was divided through the midline and the mass of each hemisphere (ipsilateral to injury) was measured. Each hemisphere was then homogenized and allowed to incubate overnight in 0.9 mL of formamide (Sigma Aldrich, St. Louis, MO, USA) at 50°C to allow for dye extraction. After centrifugation, 100 μL of the supernatant from each sample was transferred to a 96-well plate (in triplicate) and absorbance was measured at 620 nm using a VersaMax plate reader (Molecular Devices Inc., Sunnyvale, CA, USA). Samples were run in triplicate and all values were normalized to hemisphere weight.

#### Splenocyte isolation and culture

Seventy-two hours after injury, the mice underwent splenectomy with measurement of splenic mass. Next, the spleens were minced with a razor blade, washed with basic media (10% FBS and 1% penicillin/streptomycin in RPMI), crushed, and filtered through a 100 μm filter. The effluent sample from the filter was gently titurated eight to ten times and subsequently filtered through a 40 μm filter to remove any remaining connective tissue, then centrifuged at 1,000 g for 3 minutes. Next, the supernatants were removed, samples resuspended in 2 mL of red blood cell lysis buffer (Qiagen Sciences, Valencia, CA, USA) and allowed to incubate on ice for 5 minutes. Subsequently, the samples were washed twice with basic media and centrifuged using the aforementioned settings. The splenocytes were counted and checked for viability by Trypan blue exclusion (>95% viability).

#### Blood collection

At the designated time point, mice were anesthetized as described above. Blood was aspirated via left ventricular puncture and placed immediately into collection tubes with sodium heparin (BD Vacutainer, BD, Franklin Lakes, NJ, USA). Samples were placed on ice. Erythrocytes were lysed from the samples using a red blood lysis solution (BioLegend, San Diego, CA, USA). The samples were then centrifuged at 1,000 g for 3 minutes, washed in PBS and resuspended for cell staining (described below).

#### Microglia isolation and staining

The side of the brain ipsilateral to the injury was excised, minced, and mechanically disrupted using a glass homogenizer. The homogenate was washed and passed through a 40-μm cell strainer. Microglia were isolated with a discontinuous Percoll (Sigma) density gradient separation; briefly, homogenized brains were suspended in 30% Percoll in Hank’s Buffered Salt Solution (HBSS) (ρ = 1.039 g/ml), then pipetted over 70% (ρ = 1.091 g/ml) and 35% (ρ = 1.045 g/ml) Percoll layers and centrifuged at 1,000 g for 30 minutes at 20°C. Cells were removed from the 30%/35% and 35%/70% interfaces, washed twice in 10% fetal bovine serum (FBS) in HBSS, and resuspended in staining buffer (4% FBS in PBS) for flow cytometric analysis.

Samples were incubated for 20 minutes with a CD16/32-Fc receptor blocker (BioLegend, San Diego, CA, USA) to reduce nonspecific antibody binding. Samples were then incubated for 30 minutes with surface markers CD45-APC and CD86-PE (BioLegend, San Diego, CA, USA), washed with staining buffer, and resuspended in ice cold 4% paraformaldehyde in PBS. Samples were incubated for 30 minutes, then washed and resuspended in staining buffer with 0.1% Triton X-100 for another 30 minutes. Finally, samples were stained with CD206-FITC (Biolegend, San Diego, CA, USA) for 30 minutes. All incubations were performed in the dark at room temperature. Appropriate isotype controls were prepared.

#### CD4^+^/CD25^+^/FOXP3^+^ T regulatory cell staining

Peripheral blood and splenocytes from the study animals were stained using a FOXP3 T regulatory cell staining kit as per manufacturer’s suggested protocol. (BioLegend, San Diego, CA, USA). Briefly, erythrocytes were lysed from the samples using a red blood lysis solution (BioLegend, San Diego, CA, USA). The samples were then washed, resuspended, and incubated with CD25-PE and CD4-FITC for 30 minutes. Samples were then washed, resuspended, and incubated with fixation/permeabilization buffer for 30 minutes; this was followed by permeabilization buffer for 15 minutes and then incubation with FOXP3-AlexaFluor647 for 30 minutes. All incubations were performed at room temperature. Appropriate isotype controls were prepared.

### *In vitro* experiments

#### Experimental design

**Figure 2:**
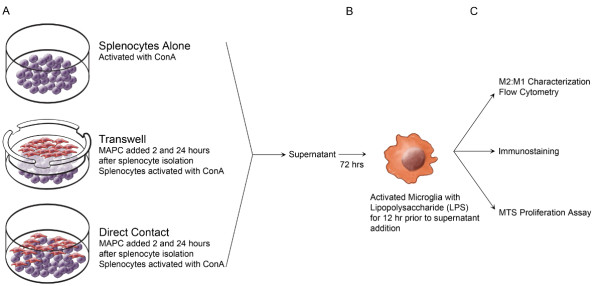
**Schematic setup of *in vitro *MAPC, splenocyte, and microglia/macrophage interactions.** (**A**) Isolated splenocytes are activated with 2 μg/ml of ConA. Two hours after isolation, MAPC were added at a ratio of 1:5 (MAPC:splenocyte) in co-culture or transwell cultures. 24 hours after the first dose of MAPCs, a second dose of MAPCs were added at the same ratio. 48 hours after the splenocyte harvest, supernatants were collected from each specific condition. (**B**) The supernatant was then used to stimulate LPS-activated microglia. (**C**) Microglia/macrophages were characterized via flow cytometry, immunostained or measured for proliferation using the MTS proliferation assay. LPS, lipopolysaccharide; MAPCs, multipotent adult progenitor cells; MTS.

We utilized cell culture techniques to delineate whether supernatant derived from direct contact between MAPCs and splenocytes (activated by mitogenic plant lectin Concanavalin A (ConA)) is required to attenuate the proinflammatory response of activated microglia (Figure 2). Briefly, we used supernatant derived from MAPC:splenocyte co-cultures to attenuate the bacterial endotoxin lipopolysaccharide (LPS) activated microglia. We then measured proliferation and apoptosis of M1 and M2 subtype microglia/macrophages.

#### MAPC:splenocyte direct contact (co-culture) and transwell cultures

**Figure 3:**
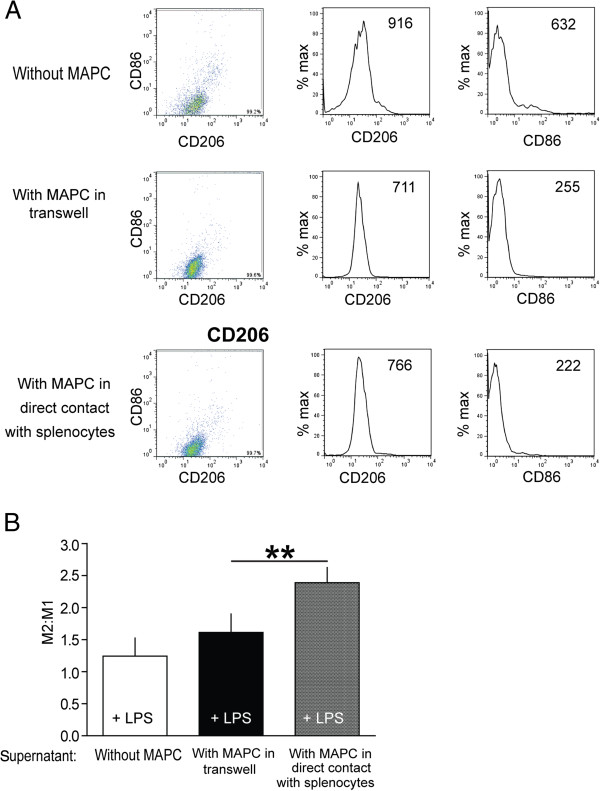
**Effect of MAPC:Splenocyte co-culture supernatant on stimulated microglial immunophenotype.** (**A**) Samples of microglial cultures were first gated on forward- and side-scatter characteristics to exclude debris, electronic noise, and aggregates (not shown). Resulting populations were then gated to exclude a small number of artifacts displaying extremely high signal for either CD206-AF488 or CD86-PE. Rather than divide this microglia population further by imposing strict boundaries on a continuous expression pattern, mean fluorescence intensity (MFI, listed in each box) of the entire remaining population was then determined for each marker and the CD206/CD86 MFI ratio (M2:M1 ratio) calculated. (**B**) There was a significant increase in the M2:M1 ratio when the microglia received the supernatant derived from MAPC:splenocyte co-culture plus LPS when compared to microglia which only received LPS alone, or supernatant derived from MAPC:splenocyte transwell cultures plus LPS. ** represents *P* <0.01. LPS, lipopolysaccharide; MAPC, multipotent adult progenitor cell.

Splenocytes were harvested from naive adult mice (n = 11/group) as described above, immediately placed into culture, and stimulated with 2 μg/ml of ConA. MAPC were added at a ratio of 1:1, 1:5, and 1:10 (MAPC:splenocyte) 2 and 24 hours after splenocyte isolation and culture, to mimic the *in vivo* tail vein injection of MAPC, in either direct contact (co-culture) or transwell cultures. There were no differences in the effects between 1:1 and 1:5 with very little effect at 1:10 (data not shown); therefore a 1:5 MAPC:splenocyte ratio was used for all experiments. Finally, 48 hours after splenocyte isolation, the supernatant was removed for use in *in vitro* microglial cultures (see Figure 3).

#### Microglial cultures

Microglia were isolated from sham/uninjured brains using the protocol described above. After isolation, microglia were grown for a month in microglial growth media which consisted of the following: Dulbecco’s modified Eagle’s/F12 medium with GlutaMAX (DMEM/F12) supplemented with 10% FBS, 100units/ml penicillin, 100 g/ml streptomycin and 5 ng/ml of granulocyte macrophage colony stimulating factor (GM-CSF) (415-ML-010/CF; R&D Systems, Minneapolis, MN, USA). After reaching confluency, cells were split into multiple-well plates and activated with 1 μg/ml bacterial endotoxin lipopolysaccharide (LPS) for 12 hours before MAPC:splenocyte co-culture supernatant was added. The volume ratio of supernatant to microglia culture was 1:4. Finally, cells were harvested after 72 hours incubation and characterized (M2 *versus* M1) by flow cytometry as described previously in the methods section (Figure 3B and 3C).

#### Immunostaining

To delineate whether microglia/macrophages are changing immunophenotypes due to injury and subsequent MAPC treatment, microglia were grown in 8-well chamber slides as described above. LPS was added 12 hours prior to the addition of either supernatant derived from splenocytes alone or MAPC:splenocyte co-culture. After 72 hours, the cells were fixed with 4% paraformaldahyde (PFA), washed three times with tris-buffered saline (TBS) (10 minutes), and then blocked at room temperature for 1 hour with 5% fetal bovine serum plus 0.25% Triton X-100 in TBS. They were then incubated with primary antibodies (CD86 (1:250; ab53004; Abcam, Cambridge, MA, USA) and CD206 (1:250; ab8918; Abcam, Cambridge, MA, USA)) in 0.25% Triton X-100 in TBS overnight at 4°C. Slidewells were then washed three times (10 minutes) at room temperature with TBS. Secondary antibodies were used at a concentration of 1:1,000 in 0.25% Triton X-100 in TBS (CD206-AlexaFluor488, A11034 and CD86-AlexaFluor568, A11004; Invitrogen, Carlsbad, CA, USA); samples were incubated for 3 hours in the dark. Cells were then washed three times (10 minutes) with TBS, mounted, and cover-slipped with Fluoromount-G (Southern Biotech, Birmingham, AL, USA). We counted the number of cells from three different frames from six different wells from microglia/macrophages exposed to supernatant from splenocyte culture alone (n = 6) and seven different wells (three frames/well) from microglia/macrophages incubated with supernatant derived from MAPC:splenocyte co-culture (n = 7). We counted the total number of cells in each well (three frames at 20x) that stained for M1 and M2, M1 only, or M2 only.

In order to determine if supernatant from MAPC:splenocyte co-cultures results in apoptosis of microglia/macrophages, we used LPS-activated microglia with and without supernatant treatment (as described above). Cells were immunostained with CD206 (1:250; ab8918; Abcam, Cambridge, MA, USA), CD86 (1:100; 550542; BD Pharmingen, San Jose, CA, USA) and cleaved caspase 3 (CC3), an apoptotic marker (1:1,500; 9664; Cell Signaling, Beverly, MA, USA). Secondary antibodies were used at a concentration of 1:1,000 in 0.25% Triton X-100 in TBS (CD206-AlexaFluor488, A11029, CD86-AlexaFluor350, A21093 and CC3-AlexaFluor568, A11011; Invitrogen, Carlsbad, CA, USA). Incubation periods, washes, and mounting were done as described above. We counted the total number of cells from eight different wells (seven frames/well at 10x magnification) from microglia/macrophages incubated with supernatant derived from MAPC:splenocyte co-cultures (n = 8) and from microglia/macrophages exposed to splenocytes alone (n = 8). We compared the number of double positive (M1/CC3 or M2/CC3) cells and the number of triple positive cells between these two groups.

#### MTS proliferation assay

In order to determine whether supernatant derived from MAPC:splenocytes co-cultures results in the attenuation of microglia proliferation, we utilized the CellTiter 96™ Aqueous Non-Radioactive Cell Proliferation Assay (Promega, Madison, WI, USA). Microglia were activated with LPS for 12 hours prior to the addition of supernatant derived from MAPC:splenocyte co-cultures or splenocyte alone cultures. After 72 hours, the cell proliferation assay was performed. Briefly, 3-(4,5-dimethylthiazol-2-yl)-5-(3-carboxymethoxyphenyl)-2-(4-sulfophenyl)-2 H-tetrazolium, inner salt (MTS) was added to 96-well plates at a ratio of 20 μl combined MTS/PMS solution per 100 μl culture medium. After the addition of MTS, the plates were incubated at 37°C for one hour and the plates were subsequently read on the VersaMax plate reader (Molecular Devices Inc., Sunnyvale, CA, USA) at 490 nm absorbance.

#### Flow cytometry

Data was acquired using a LSR II flow cytometer (BD Biosciences, San Jose, CA, USA) and analyzed using FACSDiva software (BD Biosciences, San Jose, CA, USA). Unless otherwise indicated, 10,000 events of the gated population of interest were collected for analysis.

#### Data analysis

Unless otherwise indicated, all values are represented as mean ± SEM. Values were compared using analysis of variance (ANOVA) with a post-hoc Dunnet’s test. If only two groups were compared, either an unpaired *t*-test or the Mann–Whitney test was used, as indicated. A *P* value of ≤0.05 was used to denote statistical significance.

## Results

### MAPC treatment after injury attenuates blood brain barrier permeability

**Figure 4:**
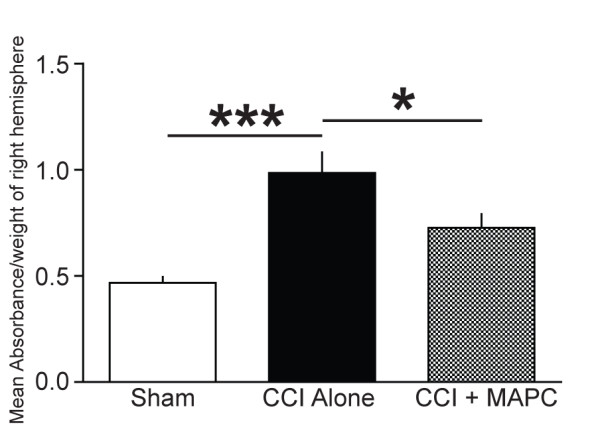
**MAPC treatment reduces BBB permeability after TBI.** BBB permeability measurement was completed using Evan’s blue dye as mean absorbance (nm) normalized to tissue weight (grams) derived from homogenized cortical tissue derived from the hemisphere ipsilateral to the CCI injury. Mice showed an increase in BBB permeability after CCI alone that was reversed by the intravenous injection of MAPC. * represents *P* <0.05 and *** represents *P* <0.005. BBB, blood brain barrier; MAPC, multipotent adult progenitor cell; TBI, traumatic brain injury.

BBB permeability measurement was completed using Evan’s Blue dye in all three groups [sham (n = 5), CCI alone (n = 6) and CCI plus MAPC (n = 6)]. Figure 4 demonstrates the mean absorbance (nm) normalized to tissue weight (grams) derived from homogenized cortical tissue from the hemisphere ipsilateral to the CCI injury. The cerebellum was not included in the measurement. Mice showed an increase in BBB permeability after CCI alone (0.98 ± 0.09) that was reversed by the intravenous injection of MAPC (0.72 ± 0.06, *P* <0.05*,* CI 0.006 to 0.523). In addition, there were significant differences between CCI alone (0.98 ± 0.09) and sham (0.46 ± 0.03, *P* <0.01, CI 0.257 to 0.518). There were no significant differences between sham and CCI plus MAPC (CI −0.002 to 0.259).

### MAPC treatment after injury preserves splenic mass

Seventy-two hours after CCI injury, mice (n = 18/group) were euthanized and spleens harvested and weighed. A significant decrease in mass (*P* ≤0.01*,* CI 0.005 to 0.033 grams) is observed in the CCI alone (0.075 ± 0.003 grams) (range 0.044 to 0.098 grams) when compared to CCI plus MAPC (0.094 ± 0.004 grams) (range 0.690 to 0.137 grams). There was no difference (*P* = 0.15*,* CI 0.005 to 0.023 grams) between sham (0.084 ± 0.006 grams) (range 0.055 to 0.150 grams) and CCI plus MAPC groups.

### MAPC treatment after injury increases T regulatory cells in spleen and peripheral blood

**Figure 5:**
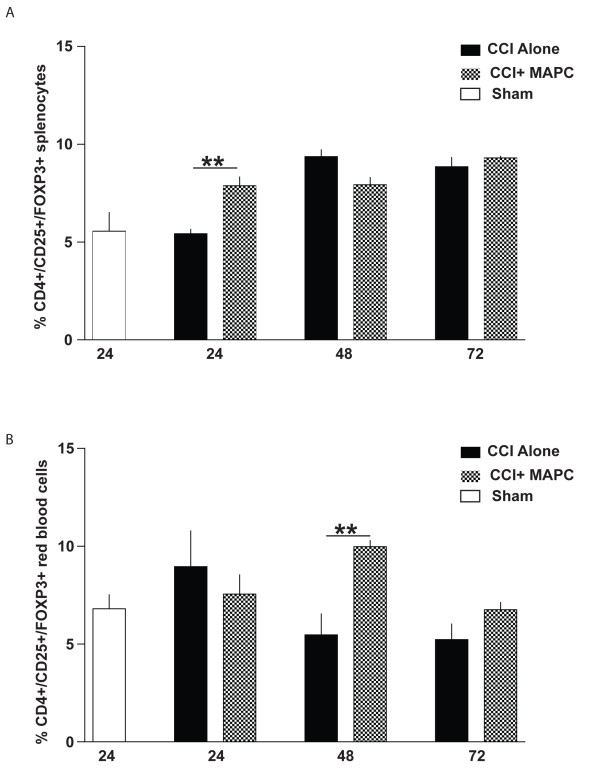
**MAPC treatment increases CD4**^**+**^**/CD25**^**+**^**/FOXP3**^**+ **^**cells after TBI in spleen and blood.** (**A**) Measurement of CD4^+^/CD25^+^/FOXP3^+^ cells was completed in the splenocytes at 24, 48, and 72 hours after CCI injury and CCI injury plus MAPC (n = 4/group). There was a significant increase in T regulatory cells as a percentage of CD4+ T helper cells within the spleens of MAPC treated mice at 24 hours (*P* ≤0.01), with no difference seen at 48 or 72 hours. (**B**) In peripheral blood, percent T regulatory cells from MAPC treated mice was significantly higher at 48 hours after CCI injury (*P* ≤0.01). Of note, there is an additional control of sham (uninjured: white bar) for baseline profile of splenocytes. * represents *P* <0.05 and ** represents *P* <0.01. CCI, controlled cortical impact; MAPC, multipotent adult progenitor cell; TBI, traumatic brain injury.

CD4^+^/CD25^+^/FOXP3^+^ T regulatory cells were characterized in the spleen and blood of mice at 24, 48, and 72 hours after CCI injury (n = 4/group). There was a significant increase in T regulatory cells as a percentage of CD4+ T helper cells within the spleens of MAPC treated mice at 24 hours (*P* ≤0.01), with no difference seen at 48 or 72 hours (Figure 5A). In peripheral blood, percent T regulatory cells from MAPC treated mice was significantly higher at 48 hours after CCI injury (*P* ≤0.01) (Figure 5B).

### Microglia/macrophages activation phenotype is influenced by MAPC treatment after injury

**Figure 6:**
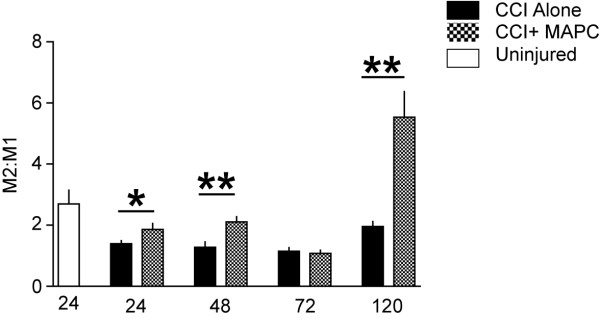
**MAPC treatment changes macrophage/microglia phenotype after TBI.** Graph of the ratio of anti-inflammatory microglia *versus* proinflammatory microglia as determined by flow cytometry. Measurement of brain CD86^+^ M1 and CD206^+^ M2 macrophages was completed after cortical injury in mice at 24, 48, 72, and 120 hours after CCI injury (n = 6/group). There were significant increases in the ratio of M2 *versus* M1 mean fluorescence intensity between the CCI plus MAPC *versus* CCI alone at 24 (*P* ≤0.05), 48 (*P* ≤0.01) and 120 hours (*P* ≤0.01) after injury* represents *P* <0.05 and ** represents *P* <0.01. CCI, controlled cortical impact; MAPC, multipotent adult progenitor cell; TBI, traumatic brain injury.

Microglia/macrophages were harvested from the brain after cortical injury in mice at 24, 48, 72, and 120 hours after CCI injury (n = 6/group). There were significant increases in the ratio of M2 *versus* M1 mean fluorescence intensity between the CCI plus MAPC *versus* CCI alone at 24 (*P* ≤0.05), 48 (*P* ≤0.01) and 120 hours (*P* ≤0.01) after injury (Figure 6).

### Soluble factor(s) modulate microglia phenotype

**Figure 7:**
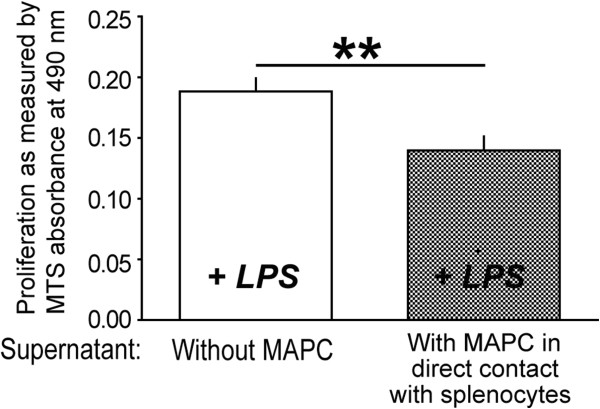
**Supernatant derived from MAPC:splencocyte co-cultures attenuates LPS induced proliferation.** There was a significant reduction in proliferation of microglia as determined by absorbance of MTS (*P* ≤0.01: n = 8) in the microglia treated with supernatant derived from MAPC:splenocyte co-culture when compared to splenocyte only culture. ** represents *P* <0.01. LPS, lipopolysaccharide; MAPC, multipotent adult progenitor cell; MTS.

In order to determine whether direct contact of MAPC with splenocytes was required to regulate macrophage phenotype, we evaluated the effect of supernatant derived from direct co-culture *versus* transwell cultures on activated microglia differentiation. After 72 hours of incubation with supernatant derived from the coculture or transwell cultures, the populations of CD86^+^ M1 macrophages and CD206^+^ M2 macrophages were measured using flow cytometry. There was a significant increase in the M2:M1 ratio when the microglia received the supernatant (2.4 ± 0.23) derived from MAPC:splenocyte co-culture plus LPS when compared to microglia which only received LPS alone (1.24 ± 0.28; *P* ≤0.05: Figure 7), or supernatant derived from MAPC:splenocyte transwell cultures plus LPS (1.61 ± 0.28).

### Soluble factor(s) attenuate microglia proliferation

**Figure 8:**
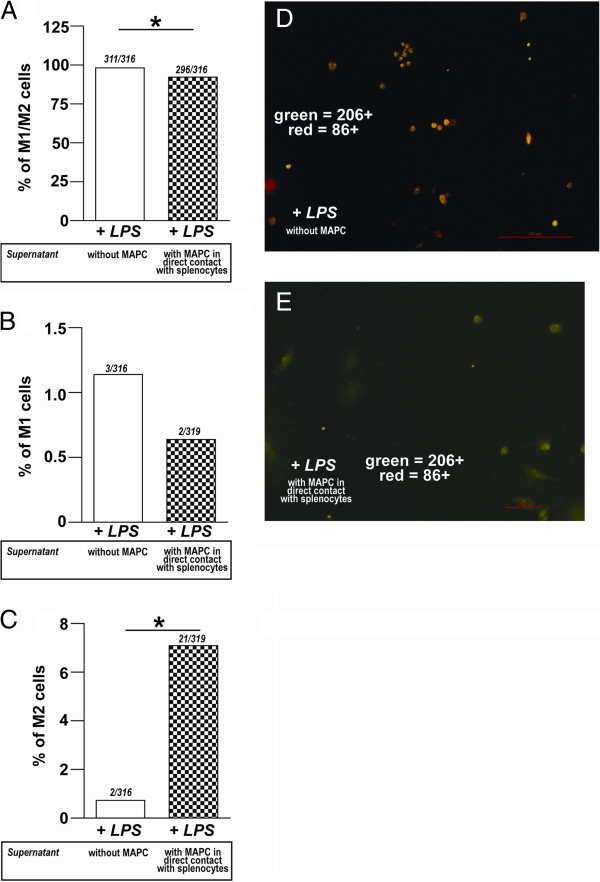
**Supernatant derived from MAPC:splencocyte co-cultures promotes anti-inflammatory phenotype of microglia/macrophages.** (**A**) Graph of percentage of M1/M2 activated with LPS and incubated with MAPC:splenocyte co-culture supernatant *versus* splenocyte culture supernatant (without MAPC). A significant decrease in the double positive microglial cells (CD86^+^ and CD206^+^) is seen in the MAPC:splenocyte co-culture group (*P* ≤0.05). (**B**) Fewer CD86^+^ microglia were observed in the MAPC:splenocyte co-culture group, but this change was not significant. (**C**) Significantly more CD206^+^ cells were observed in the MAPC:splenocyte co-culture group as opposed to the splenocyte alone group (*P* ≤0.05*).* (**D**) Photomicrograph of isolated microglia/macrophages activated LPS and splenocyte alone (without MAPC) supernatant. Note the number of dual stained cells. (**E**) Photomicrograph of isolated microglia/macrophages activated LPS and incubated with MAPC:splenocyte co-culture supernatant treatment. Note the number of M2 cells (green). M2 cells are labeled green and M1 cells are labeled red. * represents *P* <0.05. LPS, lipopolysaccharide; MAPC, multipotent adult progenitor cell.

We next evaluated whether the increase in the M2:M1 ratio was secondary to alteration in proliferation. There was a significant reduction in proliferation of microglia as determined by absorbance of MTS (*P* ≤0.01: n = 8, Figure 8) in the microglia treated with supernatant derived from MAPC:splenocyte co-culture when compared to splenocyte only culture.

### Soluble factor(s) increase anti-inflammatory microglia/macrophages

**Figure 9:**
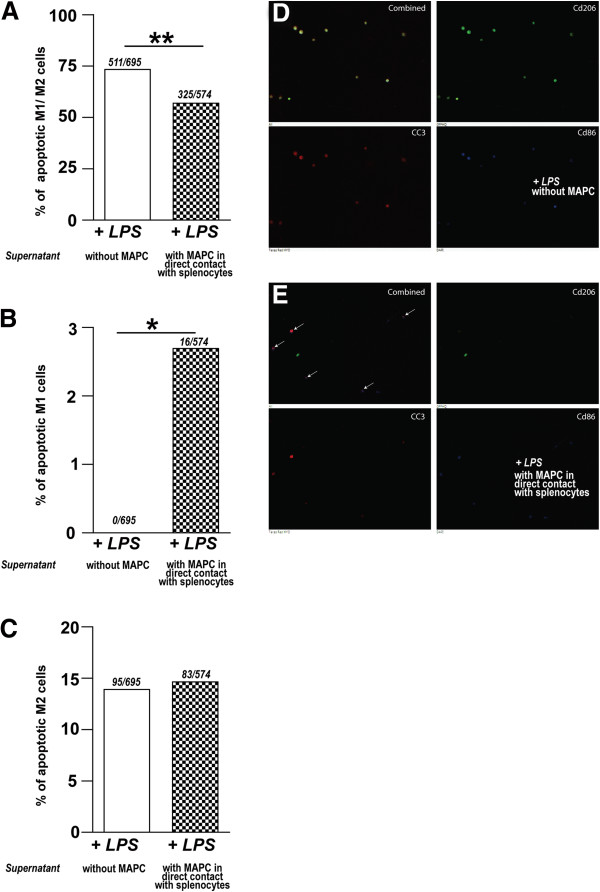
**Supernatant derived from MAPC:splenocyte co-cultures promotes apoptosis of proinflammatory microglia/macrophages.** (**A**) Triple positive microglia (CD86^+^/CD206^+^/CC3^+^) significantly decreased after treatment with supernatant derived from MAPC:splenocyte co-cultures (*P* ≤0.01). (**B**) A significant increase in M1 microglial apoptosis was measured by the number of CD86^+^/CC3^+^ microglia in microglia treated with supernatant derived from MAPC:splenocyte co-cultures (*P* ≤0.05). (**C**) No significant difference in the number of CD206^+^/CC3^+^ microglia between the two groups was observed*.* (**D**) Photomicrograph of isolated microglia/macrophages activated LPS and splenocyte alone (without MAPC) supernatant. Note the number of triple stained cells. (**E**) Photomicrograph of isolated microglia/macrophages activated LPS and incubated with MAPC in direct contact with splenocytes supernatant treatment. Note the number of M1/CC3 as indicated by arrows. M2 positive cells are labeled green, CC3 positive cells are labeled red, and M1 positive cells are labeled blue * and ** represents *P* <0.05. LPS, lipopolysaccharide; MAPCs, multipotent adult progenitor cells.

Next, we wanted to determine whether microglia/macrophages were changing their immunophenotype due to supernatant derived from MAPC:splenocyte co-culture or supernatant from splenocyte cultures alone. Most of the cells were positively labeled for both (>90% in both groups), but there was a significant decrease in the double positive microglial cells (CD86^+^ and CD206^+^) in the MAPC:splenocyte co-culture group (splenocyte alone: 98.1 ± 0.75 *versus* MAPC:splenocyte co-culture: 92.3 ± 2.2, *P* ≤0.05*,* Figure 9A, D*)*. There were significantly more CD206^+^ cells in the MAPC:splenocyte co-culture group as opposed to the splenocyte alone group (splenocyte alone: 0.73 ± 0.48 *versus* MAPC:splenocyte co-culture: 7.1 ± 2.1, *P* ≤0.05*,* Figure 9C,E). There were less CD86^+^ microglia in the MAPC:splenocyte co-culture group, but this change was not significant (splenocyte alone: 1.1 ± 0.8 *versus* MAPC:splenocyte co-culture: 0.6 ± 0.4, Figure 9B).

### Soluble factor(s) increase apoptosis of proinflammatory microglia/macrophages

There was a significant decrease in the total apoptosis when cells were treated with supernatant from MAPC:splenocyte co-cultures. Triple positive microglia (CD86^+^/CD206^+^/CC3^+^) significantly decreased after treatment with supernatant derived from MAPC:splenocyte co-cultures (splenocyte alone: 60.2 ± 3.7 *versus* MAPC:splenocyte co-culture: 43.9 ± 2.7, *P* ≤0.01*,* Figure 3A,D*)*. There was a significant increase in M1 microglial cell apoptosis as measured by the number of CD86^+^/CC3^+^ microglia in microglia treated with supernatant derived from MAPC:splenocyte co-cultures (splenocyte alone: 0.0 ± 0.0 *versus* MAPC:splenocyte co-culture: 2.7 ± 1.0, *P* ≤0.05*,* Figure 3B,E). There was no significant difference in the number of CD206^+^/CC3^+^ microglia between the two groups (splenocyte alone: 13.9 ± 2.8 *versus* MAPC:splenocyte co-culture: 14.6 ± 8.8, *P* = 1*,* Figure 3C).

## Discussion

Our data show that the neuroprotective effect of intravenous MAPC therapy is associated with an alteration in the ratio of brain microglia phenotypes from neuroinflammatory to neuroprotective. Localization of MAPCs within the spleen with concordant preservation of splenic mass prior to the observed alteration of the innate immune response points to the pivotal role of MAPC:splenocyte interaction. The time course studies of T-regulatory cells agree with *in vitro* characterizations to support the concept that soluble factors dependent upon MAPC:splenocyte direct contact influence the appearance of T regulatory cells. Known temporal and anatomic anti-inflammatory stimuli provide insight into association of these potential mechanisms of action of the observed MAPC mediated neuroprotection.

Activated microglia/macrophages have been shown to be important mediators of injury after TBI [15]. Previously, we have shown that the intravenous delivery of MAPC after TBI preserved the BBB, preserved splenic mass, and increased anti-inflammatory cytokine production (IL-4 and IL-10). Interestingly, an increase in the number and proliferation of CD4^+^ T cells was found to be present in the spleens of treatment animals [11]. This pattern of findings led us to evaluate a potential role for CD4^+^/CD25^+^/FOXP3^+^ T-regulatory cells, a subtype of CD4^+^ T cells with known anti-inflammatory properties, as being potential mediators of neuroprotection. In addition, we have previously shown that in the absence of a spleen (surgical splenectomy prior to injury), TBI did not induce a severe breach in the BBB as compared to the animals who did not undergo pre-injury splenectomy. Our hypothesis was also bolstered by recent work demonstrating a potential protective role of T-regulatory cells in stroke and neurodegenerative diseases (S. Savitz *et al.* unpublished observations) and reports suggesting that T regulatory cells may work in part by altering macrophage phenotype [16]. In these experiments we have demonstrated that the increase in splenic T-regulatory cells correlated with a shift in the post-TBI brain microglia phenotype to a predominantly anti-inflammatory (M2) subtype (Figures 3, 5 and 7A,C).

**Figure 10:**
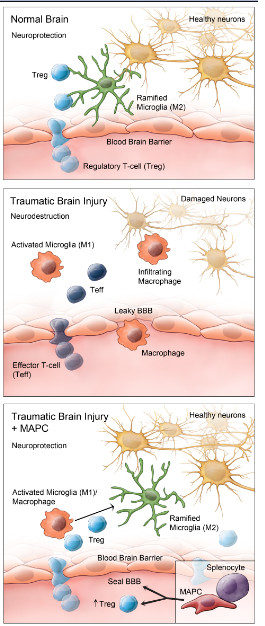
**Model of MAPC interactions with splenocytes to modulate microglia/macrophage phenotype.** (**A**) In a normal uninjured brain resident microglia generally exist in a ramified M2 state. Functions include patrolling the brain to detect any disturbances or foreign substances as well as many other functions. This allows for a neuroprotection of neurons and other cell types. Regulatory T-cells (Treg) are in constant communication with the resident ramified microglia (**B**) After TBI, ramified microglia change into activated microglia, which function mainly to phagocytose cellular debris created after injury. In addition to the activated resident microglia, there is influx of macropahges due to the damaged (leaky) BBB as well as upregulation of chemoattractant molecules and adhesion factors. After injury activated microglia and infiltrating macrophages are indistinguishable from each other. Both these cell types are being modulated by effector T-cells (Teff). (**C**) After TBI, MAPC treatment helps seal the leaky BBB. In addition, with direct contact with splenocytes, MAPC treatment results in Treg proliferation. This in turn aids in modulating the activated microglia/macrophage into ramified microglia, thereby reducing the long-term proinflammatory effects of activated microglia. BBB, blood brain barrier; MAPC, multipotent adult progenitor cell; TBI, traumatic brain injury.

Fischer *et al.* demonstrated that intravenously injected progenitor cells initially were trapped in the spleen in addition to the lungs [17]. Subsequently, Walker *et al.* traced intravenously injected MAPC to the white pulp of the spleen in close approximation with the blood vessels suggesting an interaction of MAPC with the resident splenic lymphocyte population [11]. The *in vivo* data demonstrates an initial increase in splenic T-regulatory cells at 24 hours following intravenous injection of MAPC (Figure 5A), further supporting the notion of direct interaction of splenocytes and MAPC. In addition, our *in vitro* data supports direct contact between splenocytes and MAPC as necessary for modulation of the microglia/macrophages towards an anti-inflammatory phenotype (see Figure 10).

In a non-injured brain microglia could be regulated by T regulatory cells (Figure 3A). After traumatic brain injury, there is activation of resident microglia and infiltration of macrophages due to the damaged blood brain barrier, similar to an injured spinal cord [7]. This may be directed by proinflammatory effector T cells (Figure 10B). The main focus of these activated proinflammatory myeloid cells is to phagocytose damaged cells such as neurons and clear other cellular debris. The infiltrating macrophages could be activated within the vasculature and/or in the injured microenvironment with complex interactions among effector T cells and resident microglia [18]. In addition to activation from a resting state, resident microglia/macrophages could also be proliferating. Our *in vitro* results presented here demonstrate that microglia proliferate after LPS stimulation (Figure 9), and proliferation is known to be associated with the proinflammatory phenotype of microglia/macrophage.

With MAPC treatment, we observe a significant overall reduction in proliferation of microglia/macrophages (Figure 8). MAPC:splenocyte derived supernatant treatment also decreases overall apoptosis while significantly increasing apoptosis of M1 microglia (Figure 3B) as measured by cleaved caspase 3. In addition to an increase in apoptosis of proinflammatory M1 microglia, we also observed an increase in proliferation of the anti-inflammatory M2 phenotype (Figure 3C) with MAPC treatment. Thus MAPC treatment (via soluble factors) influences the microglia/macrophages towards an overall anti-inflammatory direction in culture. *In vivo*, MAPC treatment results in a significant increase of CD4^+^/CD25^+^/FOXP3^+^T regulatory cells after 24 hours in the spleen (Figure 5A) and 48 hours in the blood (Figure 5B). Further *in vivo* characterization of local microglial apoptosis/proliferation and the pathways and mediators involved is an important next step in clarifying the mechanism of progenitor cell mechanism after TBI. Expansion and deployment of these cells could be important in earlier mitigation of proinflammatory signals closer to the site of injury via anti-inflammatory Treg cytokine secretion. Furthermore, changes in microglia/macrophage may also occur due to direct contact between T-cells and microglia/macrophages. Molecules such CD11A, CD4, CD86 and major histocompatibility complex class II molecules (MHCII) are all possible points of direct interaction between T-cells and microglia [18].

In summary, these findings establish the importance of cell therapy to systemically attenuate microglia/macrophage-induced inflammation after TBI. Our data show that the intravenous injection of bone marrow-derived MAPC increases the percentage of T regulatory cells within the spleen. The observed increased in T regulatory cells could potentially modulate the systemic inflammatory response leading to preferential differentiation of microglial/macrophage cells into the neuroprotective M2 phenotype. The *in vitro* data support the concept that soluble factors influenced by MAPC:splenocyte interactions affect microglia/macrophages by decreasing overall proliferation and apoptosis, by specifically increasing apoptosis of proinflammatory microglia and converting microglia/macrophages towards an anti-inflammatory phenotype. Overall, we believe that our data again confirm the central role of splenocytes in the observed neuroprotection seen with progenitor cell therapy, thereby providing further evidence that the transplanted cells afford benefit via a systemic rather than localized effect.

## Abbreviations

BBB: Blood brain barrier; CCI: Controlled cortical impact; DMEM/F12: Dulbecco’s modified Eagle’s/F12 medium with GlutaMAX; GM-CSF: Granulocyte macrophage colony stimulating factor; HBSS: Hank’s Buffered Salt Solution; hMAPC: Human multipotent adult progenitor cells; LPS: Lipopolysaccharide; MAPC: Multipotent adult progenitor cells; MTS: 3-(4,5-dimethylthiazol-2-yl)-5-(3-carboxymethoxyphenyl)-2-(4-sulfophenyl)-2 H-tetrazolium salt; PFA: Paraformaldahyde; PBS: Phosphate buffered saline; TBI: Traumatic brain injury; TBS: Tris-buffered saline; Teff: Effector T-cells; Treg: Regulatory T-cells.

## Competing interest

There are no known conflicts between the authors and the information presented in this paper. Charles S. Cox Jr. MD has sponsored research agreements with Athersys, Inc. and Cord Blood Registry, Inc. Peter A. Walker MD, Fernando Jimenez MS, Shinil K. Shah DO, and Charles S. Cox, Jr. MD have received grant support from BD Biosciences, Inc. Jason A. Hamilton PhD and Robert W. Mays PhD are employed by Athersys, Inc. Athersys Inc. supplied the bone marrow derived progenitor cells for all experiments.

## Authors’ contributions

PW involved in the planning of experiments, completion of experiments, data analysis, and manuscript preparation. SB involved in the planning of experiments, completion of experiments, data analysis, and manuscript preparation. SS involved in the planning of experiments, completion of experiments, data analysis, and manuscript preparation. FJ involved in the planning and completion of experiments. HX involved in the completion of experiments. JH involved in the planning of experiments. PS involved in the completion of experiments. CT involved in the completion of experiments. RM involved in the planning of experiments. SP involved in the planning of experiments. CC involved in the planning of experiments, data analysis, and manuscript preparation. Dr. Cox is also the mentoring author and PI of the lab. All authors read and approved the final manuscript.

## Supported by grants

NIH T32 GM 08 79201; M01 RR 02558; Texas Higher Education Coordinating Board; Children’s Memorial Hermann Hospital Foundation; Texas Emerging Technology Fund; Athersys, Inc.; BD Biosciences.
